# Dynamic Blood Concentrations of Aβ_1–40_ and Aβ_1–42_ in Alzheimer’s Disease

**DOI:** 10.3389/fcell.2020.00768

**Published:** 2020-08-11

**Authors:** Yuan-Han Yang, Ling-Chun Huang, Sun-Wung Hsieh, Li-Ju Huang

**Affiliations:** ^1^Department of Neurology, Kaohsiung Medical University Hospital, Kaohsiung Medical University, Kaohsiung, Taiwan; ^2^Department of Neurology, Kaohsiung Municipal Ta-Tung Hospital, Kaohsiung Medical University Hospital, Kaohsiung, Taiwan; ^3^Center of Teaching and Research, Kaohsiung Municipal Hsiao-Kang Hospital, Kaohsiung Medical University, Kaohsiung, Taiwan; ^4^Department of and Master’s Program in Neurology, Faculty of Medicine, Kaohsiung Medical University, Kaohsiung, Taiwan; ^5^Neuroscience Research Center, Kaohsiung Medical University, Kaohsiung, Taiwan; ^6^Department of Neurology, Kaohsiung Municipal Hsiao-Kang Hospital, Kaohsiung Medical University, Kaohsiung, Taiwan

**Keywords:** amyloid-beta-protein, Alzheimer’s disease, APOE, CDR, dementia

## Abstract

Amyloid-beta (Aβ) is produced by the cleavage of amyloid precursor proteins in the cell membrane by β-secretase and γ-secretase into a monomeric form with peptides of different lengths such as Aβ_1–40_ or Aβ_1–42_, which is then transformed into oligomeric and fibril forms and is considered to be one of the hallmarks of Alzheimer’s disease (AD). The plasma concentrations of Aβ_1–40_ and Aβ_1–42_ are unstable after blood samples have been obtained. In order to examine the dynamic changes of plasma Aβ_1–42_ and Aβ_1–40_ in blood samples, we used fresh blood samples in ethylenediaminetetraacetic acid tubes from 32 clinically diagnosed AD patients. Each sample was subdivided into eight sub-samples, and levels of Aβ_1–40_ and Aβ_1–42_ were measured at 0 (baseline), 0.5, 1, 2, 3, 5, 8, and 24 h, respectively. All samples were incubated at 37°C before being measuring. The results showed that compared to baseline, 87.5 and 62.5% of the patients had higher plasma levels of Aβ_1–42_ and Aβ_1–40_ at 24 h, respectively. The patients with an increased amyloid level did not have a significantly different apo-lipoprotein E4 allele (APOE4) gene status for either Aβ_1–40_ (*p* = 0.422) or Aβ_1–42_ (*p* = 1.000). However, for plasma Aβ_1–42_, the APOE4 carriers had a significantly lower level than the non-carriers at baseline [31.2 ± 6.5 (mean ± SD) ng/ml vs. 50.4 ± 47.7 ng/ml, *p* = 0.031] and 0.5 h (37.5 ± 7.6 ng/ml vs. 51.9 ± 30.8 ng/ml, *p* = 0.043). There were no significant differences between the APOE4 carriers and non-carriers in plasma Aβ_1–42_ concentration at 1, 2, 3, 5, 8, and 24 h (*p* = 0.112, *p* = 0.086, *p* = 0.112, *p* = 0.263, *p* = 0.170 and *p* = 0.621, respectively). The Aβ_1–40_ level was related to disease severity as assessed using the clinical dementia rating (CDR) scale. Patients with advanced stages of dementia (CDR = 1 and CDR = 2) had a significantly higher Aβ_1–40_ level compared to those with very mild stage dementia (CDR = 0.5) at all time points (*p* < 0.05) except for 24 h (*p* = 0.059). Our findings illustrate the effects of APOE4 status on dynamic changes in plasma Aβ_1–40_ and Aβ_1–42_ levels, and significant associations between Aβ_1–40_ level and disease severity. Further studies are needed to investigate the exact mechanisms of how APOE4 affects the dynamic changes in plasma Aβ_1–40_ and Aβ_1–42_, and the association between Aβ_1–40_ and advanced dementia.

## Introduction

As people age, the aging process, cardiovascular disease (Prince, 2015; Yang et al., 2018) and other factors (Winblad et al., 2016; Yang et al., 2019) may increase the risk of Alzheimer’s disease (AD). It has been estimated that 46.8 million people worldwide were living with dementia in 2015, and this number is expected to reach 74.7 million by 2030 and 131.5 million by 2050 (Hebert et al., 2004; Prince, 2015). AD is the most common form of dementia worldwide (Hebert et al., 2004; Prince, 2015; Yang et al., 2019). The neuropathological hallmarks of AD are formations of senile plaques composed of amyloid-beta (Aβ) peptides and neurofibrillary tangles consisting of abnormal deposition of tau protein in the brain (Karn et al., 2007; Tapiola et al., 2009). Aβ is produced by the cleavage of amyloid precursor proteins into a monomeric form with peptides of different lengths by β-secretase and γ-secretase, which is then transformed into oligomeric and fibril forms, and eventually into amyloid plaques (Haass and Selkoe, 2007; Tapiola et al., 2009) in brain tissue. Aβ_1–42_ and Aβ_1–40_ in cerebrospinal fluid (CSF) are regarded to be biomarkers in the diagnosis of AD (Strozyk et al., 2003; Tapiola et al., 2009), although consensus with regards to the standard procedures for detecting Aβ_1–42_ and Aβ_1–40_ concentrations in CSF is currently lacking. Moreover, given the invasiveness of obtaining CSF for examinations and inter-laboratory variability in the detection of Aβ_1–42_ and Aβ_1–40_ concentrations (Mattsson et al., 2010, 2012, 2013), CSF examinations for Aβ_1–40_ and Aβ_1–42_ level are not always practical.

To overcome this problem, many researchers have focused on identifying blood-based biomarkers for AD, however the results have been inconsistent (van Oijen et al., 2006). van Oijen et al. (2006) reported that a higher Aβ_1–40_ concentration but not Aβ_1–42_ or Aβ_1–40_/Aβ_1–42_ ratio was associated with a higher risk of AD. In addition, several studies have reported that a higher plasma Aβ_1–42_ level was mildly associated with AD, but that this association was not found in subsequent longitudinal examinations of Aβ_1–40_, Aβ_1–42_ level, or its ratio (Lopez et al., 2008; Mayeux and Schupf, 2011). These discrepant results may be due to several causes. First, it is not currently known whether plasma Aβ_1–40_ or Aβ_1–42_ peptides in AD patients originate from the brain, peripheral tissues or both sources (Kuo et al., 1999). Second, variations in laboratory protocols for handling samples of Aβ_1–40_ and Aβ_1–42_, and the physicochemical properties of Aβ_1–40_ and Aβ_1–42_ peptides. However, supporting evidence for these hypotheses is currently lacking (Mattsson et al., 2011; Rissman et al., 2012).

Enzyme-linked immunosorbent assay (ELISA) has been reported to be a standardized method for the quantification of Aβ_1–40_ and Aβ_1–42_ in clinical studies as biomarkers of AD (Mattsson et al., 2012). In a comparison study of different immunoassay platforms, the Alzheimer’s Association quality-control program (Mattsson et al., 2013) reported 20–30% within- and between-laboratory variability in the quantification of Aβ_1–42_ and Aβ_1–40_. A consensus with regards to the protocol for the ELISA quantification of Aβ_1–42_ and Aβ_1–40_ is still lacking.

Confounding factors for ELISA measurements include automatic plate washing, the use of polypropylene plates for pre-incubation, and the duration of sample thawing at room temperature. ELISA usually uses samples that are thawed at room temperature before being measured, which does not accurately reflect the condition in human blood, at around 37°C. In addition, the duration of sample thawing at room temperature may be associated with changes in the concentration of amyloid peptides because the process of amyloid aggregation from monomers to oligomers or fibrils is continuous, and some peptides would degrade over time.

The aim of this study was to understand the actual status of Aβ_1–40_ and Aβ_1–42_ in human blood and the possible changes in Aβ_1–40_ and Aβ_1–42_ during the measuring process in relation to other factors such as apo-lipoprotein E gene (APOE) status (Kang et al., 2015) and disease severity. We examined the concentrations of plasma Aβ_1–40_ and Aβ_1–42_ at different time points and assessed their associations with APOE genetic status and disease severity in fresh blood samples obtained from AD patients.

## Materials and Methods

### Patients

All patients diagnosed with AD were recruited from the Department of Neurology, Kaohsiung Municipal Ta-Tung Hospital, an area hospital in southern Taiwan. The diagnosis of AD was based on the NINCDS-ADRDA criteria (McKhann et al., 2011), and involved a series of comprehensive neuropsychological tests, including the Mini-Mental State Examination (MMSE) derived from the Cognitive Abilities Screening Instrument (CASI) (Lin et al., 2002), CASI, and Clinical Dementia Rating (CDR) scale (Morris, 1991). Patients with other conditions possibly contributing to the diagnosis of AD were excluded.

### Evaluations

All procedures were approved by the Kaohsiung Medical University Hospital Institutional Review Board, and written informed consent was obtained from all participants or their legal representatives. For each recruited AD patient, a series of neuropsychological assessments, including the MMSE, CASI, and CDR, were administered every 12 months to trace the clinical outcomes. The MMSE, CASI, and CDR were conducted by a senior neuropsychologist and an experienced physician based on information from a knowledgeable collateral source (usually a spouse or adult child).

### Apolipoprotein E (APOE) Genotyping

For every AD patient, restriction enzyme isotyping of the APOE allele was performed following a modification of the protocol developed by Pyrosequencing^1^. In brief, 10 ng of DNA was amplified in a 20 μL reaction volume in which dGTP was replaced by a mixture of 25% dGTP and 75% dITP to facilitate analysis of the GC-rich fragment. A 276-bp fragment was generated using the forward primer AGA CGC GGG CAC GGC TGT and reverse biotin-labeled primer CTC GCG GAT GGC GCT GAG. Single-strand DNA was prepared using streptavidin coated beads, and APOE gene variants at codons 112 and 158 were sequenced using the following primers and dispensation order: SNP112 GAC ATG GAG GAC GTG and SNP158 CCG ATG ACC TGC AGA and dispensation order GCTGAG CTAGCGT. Individuals with one or two copies of the APOE4 allele were considered to be APOE4 positive [APOE4(+)], and otherwise APOE4 negative [APOE4(−)].

### Plasma Sample for ELISA

Venous blood was drawn by venipuncture in the morning after an overnight fast. Plasma samples were collected in ethylenediaminetetraacetic acid (EDTA) vacutainers, which were immediately centrifuged for 10 min at 3000 rpm. After centrifugation, each sample was divided into eight sub-samples and incubated at 37°C. Aβ_1–42_ and Aβ_1–40_ levels were measured in the eight sub-samples at different time points, including baseline (0 h), immediately after obtaining the blood sample (0.5 h), and then at 1, 2, 3, 5, 8, and 24 h, respectively. All samples were incubated at 37°C before being measured. An increase in Aβ_1–42_ or Aβ_1–40_ level was defined according to the difference in concentration between baseline and 24 h. If the 24-h concentration was higher/lower than the baseline level, the patient was defined as having an increase/decrease in Aβ_1–42_ or Aβ_1–40_. Quantification of Aβ_1–42_ and Aβ_1–40_ in plasma was performed using a specific ELISA kit (Human Amyloid β(1–40) Assay Kit – IBL, code number 27713; and Human Amyloid β(1–42) Assay Kit – IBL, code number 27711). All assays were performed according to the manufacturer’s protocol. All reagents were prepared at room temperature (20–25°C) approximately 30 min before use.

### Statistical Analysis

Data analysis was performed using SPSS statistical software (Standard version 11.5.0; SPSS Inc., Chicago, IL, United States). All statistical tests were two-tailed, and *p* > 0.05 was taken to indicate significance. Age, education, CASI, MMSE, Aβ_1–42_, and Aβ_1–40_ were treated as continuous variables, and sex, APOE4 status, CDR, increase in Aβ_1–40_ and increase in Aβ_1–42_ were treated as categorical variables.

Independent *t*-tests for the two independent groups [APOE4(+) and APOE4(−) groups] were used to assess differences in plasma concentrations of Aβ_1–42_ and Aβ_1–40_. Repeated measures ANOVA was used to examine differences in Aβ_1–42_ and Aβ_1–40_ across all eight sub-samples.

## Results

In total, 32 AD patients were recruited into the study. The mean (±SD) age of the patients was 77.7 ± 7.3 years, and 28.1% were APOE4(+) (Table 1). Each patient had eight samples in which plasma Aβ_1–42_ and Aβ_1–40_ levels were measured at different time points. Compared to the baseline level, 87.5 and 62.5% of the patients had an increase in plasma Aβ_1–42_ and Aβ_1–40_ levels at 24 h, respectively (Table 1). Other clinical and demographic characteristics are shown in Table 1.

**TABLE 1:** Demographic characteristics of the recruited patients.

***N* = 32**	
Age, years (mean ± SD)	77.7 ± 7.3
Education, years (mean ± SD)	8.9 ± 5.0
Female (n, %)	17, 53.1%
APOE4(+)* (n, %)	9, 28.1%
CDR stage	
0.5	11, 34.4%
1	17, 53.1%
2	4, 12.5%
CASI	55.3 ± 22.7
MMSE	16.5 ± 6.3
Increased amyloid β_1–40_^∧^ (n, %)	20, 62.5%
Increased amyloid β_1–42_^∧^ (n, %)	28, 87.5%

CDR, Clinical Dementia Rating; CASI, Cognitive Ability Screening Instrument; MMSE, Mini-mental Status Examination. *At least having an allele of apolipoprotein E4 gene. Defined as 24 h level – baseline level ≥ 0.

**FIGURE 1:**
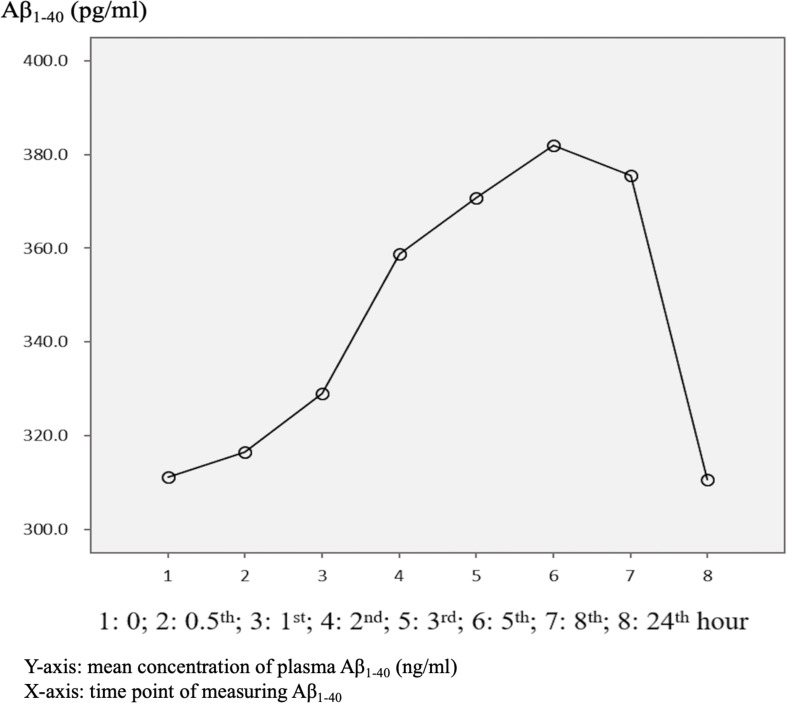
Overview of mean plasma beta-amyloid_1–40_ (Aβ_1–40_) concentration in AD patients (*N* = 32) in relation to time.

The mean baseline level of plasma Aβ_1–40_ was 314.1 ± 178.9 pg/ml, compared to 320.3 ± 157.9 pg/ml at 24 h. Overall, 62.5% of the patients had an increase in the level of plasma Aβ_1–40_ at 24 h (Table 2 and Figure 1). There was no significant difference in the ratio of AD patients having an increase in Aβ_1–40_ level between the APOE4(+) and APOE4(−) groups (*p* = 0.422).

**TABLE 2:** Plasma concentration of amyloid β_1–40_ (Aβ_1–40_) by time in the patients with Alzheimer’s disease.

	Total sample *N* = 32	*p*-value	APOE4(+)^$^ *n* = 9	APOE4(−)^#^ *n* = 23	*p*-value	CDR = 0.5 *n* = 11	CDR > 0.5 *n* = 21	*p*-value
Aβ_1–40_, pg/ml (mean ± SD)		*p* = 0.001						
**Time (h)**								
0	314.1 ± 178.9		286.4 ± 196.4	325.0 ± 175.0	0.564	201.3 ± 152.7	369.5 ± 168.5	0.010
0.5	318.5 ± 178.1		302.1 ± 183.8	324.8 ± 179.6	0.681	199.1 ± 143.1	381.0 ± 164.2	0.004
1	330.1 ± 184.2		326.2 ± 191.6	332.6 ± 185.5	0.869	219.8 ± 166.4	389.0 ± 168.5	0.011
2	361.7 ± 184.8		355.0 ± 184.9	364.3 ± 190.9	0.902	250.0 ± 159.3	420.1 ± 172.6	0.011
3	373.4 ± 189.3		359.3 ± 184.9	379.0 ± 194.8	0.837	254.8 ± 165.2	435.6 ± 173.5	0.008
5	384.5 ± 187.2		383.7 ± 199.8	384.9 ± 186.7	0.773	272.5 ± 175.6	443.2 ± 168.4	0.012
8			381.5 ± 193.9	389.5 ± 194.6	1.000	283.9 ± 171.2	441.3 ± 181.9	0.024
24	320.3 ± 157.9		268.6 ± 112.1	340.6 ± 170.4	0.386	247.8 ± 169.4	358.4 ± 141.0	0.059
Increased* (n/N, %)	20/32, 62.5%		7/9, 77.8%	13/23, 56.5%	0.422	9/11, 81.8%	11/21, 52.4%	0.104

CDR, Clinical Dementia Rating. *Defined as 24 h level – baseline level ≥ 0. ^$^At least having an allele of apolipoprotein E4 gene. ^#^No allele of the apolipoprotein E4 gene.

**FIGURE 2:**
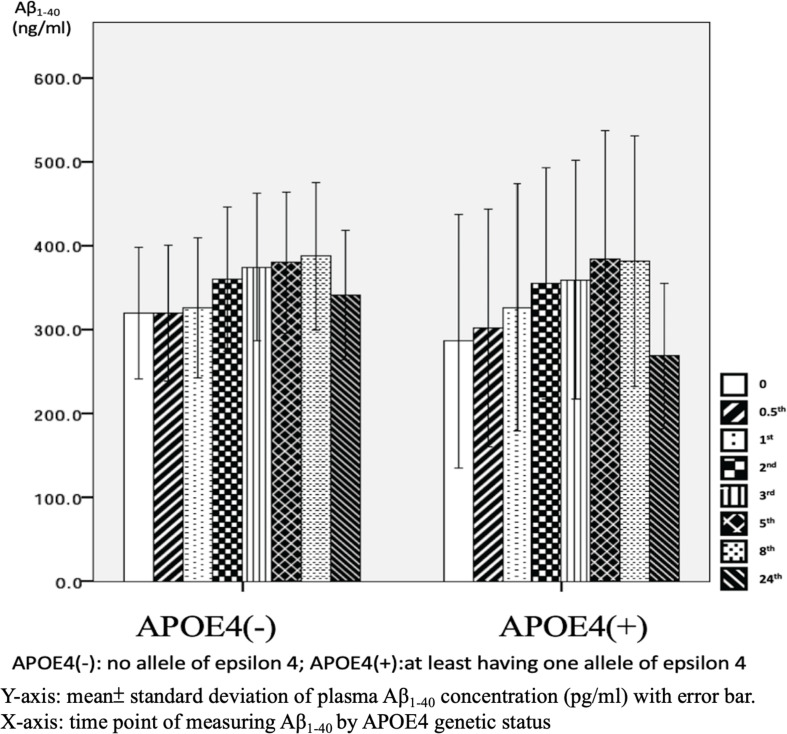
Plasma concentration of beta-amyloid_1–40_ (Aβ_1–40_) at different time point by apolipoprotein E4 (APOE4) genetic status (*N* = 32).

**FIGURE 3:**
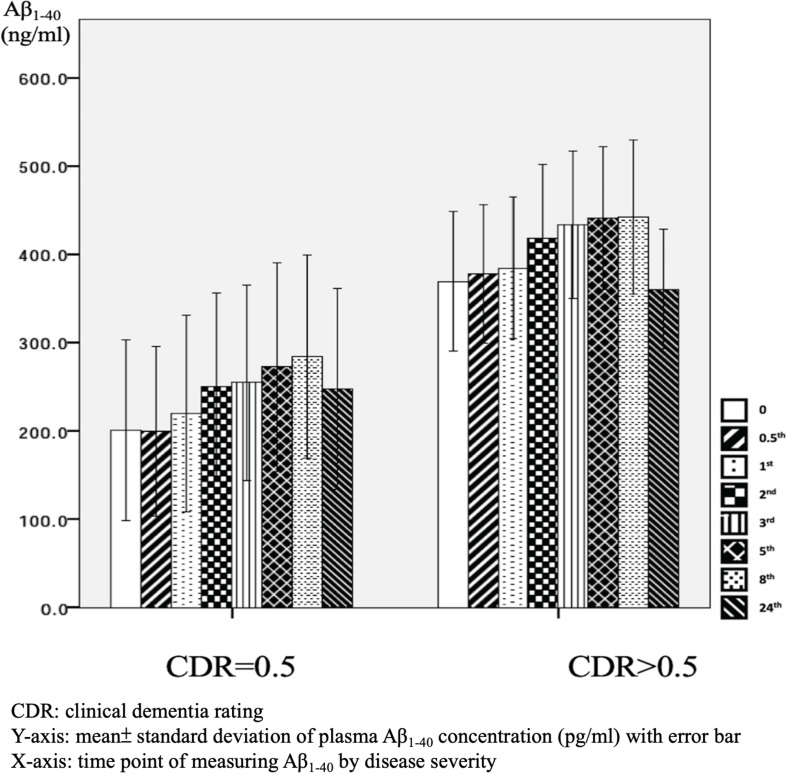
Plasma concentration of beta-amyloid_1–40_ (Aβ_1–40_) at different time point by disease severity (*N* = 32).

There were significant differences in Aβ_1–40_ level at all time points, and the concentration for any one was significantly different to the others (*p* = 0.001). However, there were no significant differences in Aβ_1–40_ level at any time point between the APOE4(+) and APOE4(−) groups (*p* = 0.386–1.000) (Table 2 and Figure 2). In addition, there were significant differences in Aβ_1–40_ level according to disease severity at all time points (*p* = 0.008–0.024) except for 24 h (*p* = 0.059) (Table 2). The patients with very mild stage dementia (CDR = 0.5) had a lower Aβ_1–40_ level compared to those with an advanced stage of dementia (CDR > 0.5) (Table 2 and Figure 3).

**FIGURE 4:**
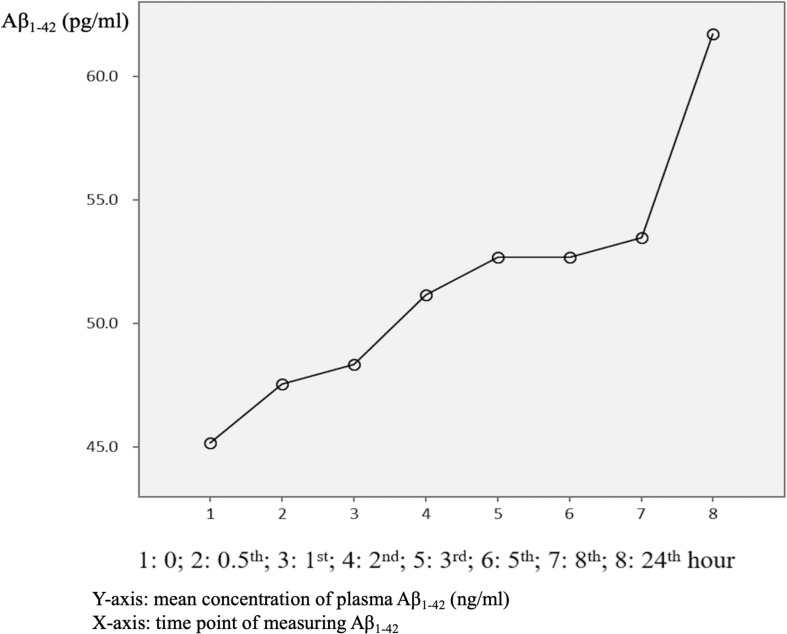
Overview of mean plasma beta-amyloid_1–40_ (Aβ_1–40_) concentration in AD patients (*N* = 32) in relation to time.

The mean baseline plasma Aβ_1–42_ level was 45.0 ± 41.3 pg/ml, compared to 63.6 ± 59.4 pg/ml at 24 h. Compared to the baseline level, 87.5% of the patients had an increase in the level at 24 h (Table 3 and Figure 4). There was no significant difference in the ratio of AD patient having an increase in Aβ_1–40_ level between the APOE4(+) and APOE4(−) groups (*p* = 1.000).

**TABLE 3:** Plasma concentration of amyloid β_1–42_ (Aβ_1–42_) by time in the patients with Alzheimer’s disease.

	Total samples *N* = 32	*p*-value	APOE4(+)^$^ *N* = 9	APOE4(−)^#^ *N* = 23	*p*-value	CDR = 0.5	CDR > 0.5	*p*-value
Aβ_1–42_ pg/ml (mean ± SD)		*p* = 0.042						
**Time (h)**								
0	45.0 ± 41.3		31.2 ± 6.5	50.4 ± 47.7	0.031	40.9 ± 28.5	47.2 ± 47.1	0.691
0.5	47.8 ± 27.1		37.5 ± 7.6	51.9 ± 30.8	0.043	44.9 ± 16.8	49.4 ± 31.4	0.663
1	48.6 ± 28.9		39.6 ± 8.2	52.1 ± 33.3	0.112	46.0 ± 13.7	49.9 ± 34.6	0.721
2	51.6 ± 26.7		42.6 ± 9.5	55.1 ± 30.5	0.086	48.7 ± 12.7	53.1 ± 31.9	0.671
3	53.0 ± 22.6		45.1 ± 9.1	56.1 ± 25.5	0.112	50.8 ± 11.4	54.2 ± 26.8	0.699
5	52.9 ± 18.2		46.9 ± 9.5	55.3 ± 20.4	0.263	52.2 ± 11.2	53.3 ± 21.3	0.867
8	55.1 ± 19.0		48.4 ± 10.3	57.8 ± 21.1	0.170	52.9 ± 9.2	56.3 ± 22.7	0.632
24	63.6 ± 59.4		60.8 ± 42.4	64.7 ± 65.7	0.621	52.6 ± 9.5	69.4 ± 72.9	0.455
Increased* (n/N, %)	(28/32, 87.5%)		8/9, 88.9%	20/23, 87.0%	1.000	9/11, 81.8%	19/21, 90.5%	0.427

CDR, Clinical Dementia Rating. *Defined as 24 h level – baseline level ≥ 0. ^$^At least having an allele of apolipoprotein E4 gene. ^#^No allele of the apolipoprotein E4 gene.

**FIGURE 5:**
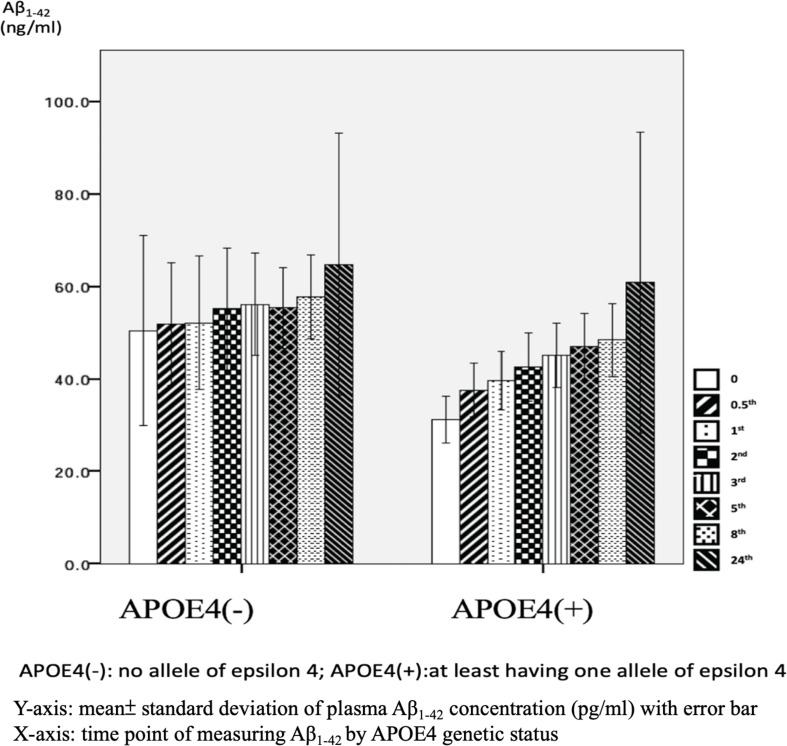
Plasma concentration of beta-amyloid_1–42_ (Aβ_1–42_) at time point by apolipoprotein E4 (APOE4) genetic status (*N* = 32).

**FIGURE 6:**
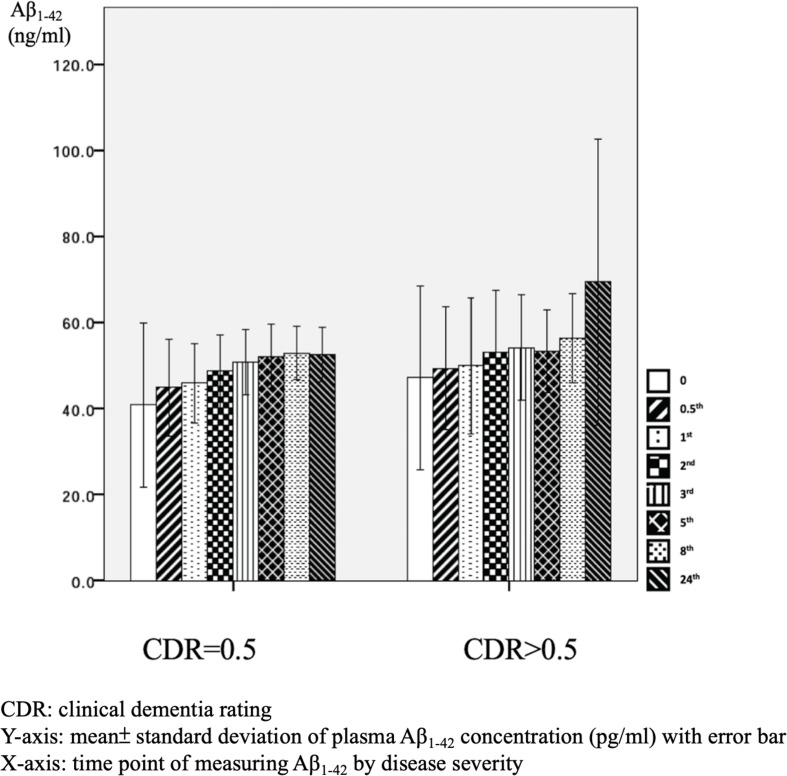
Plasma concentration of beta-amyloid_1–42_ (Aβ_1–42_) at different time point by disease severity (*N* = 32).

There were significant differences in Aβ_1–42_ level at all time points, and the concentration for any one was significantly different to the others (*p* = 0.042). There were significant differences between the APOE4(+) and APOE4(−) groups in Aβ_1–42_ level at baseline (31.2 ± 6.5 pg/ml vs. 50.4 ± 47.7 pg/ml, *p* = 0.031) and 0.5 h (37.5 ± 7.6 pg/ml vs. 51.9 ± 30.8 pg/ml, *p* = 0.043). Apart from these two time points, there were no significant differences in the other time points between the APOE4(+) and APOE4(−) groups (*p* = 0.086–0.621) (Table 3 and Figure 5). There were also no significant differences in Aβ_1–42_ level at any time point by disease severity (*p* = 0.427–0.867) (Table 3 and Figure 6). We also analyzed the associations between the Aβ_1–40_/Aβ_1–42_ ratio and APOE4 genotypes and disease severity at all time points, and the results were similar to those for Aβ_1–40_ and Aβ_1–42_ alone. Aβ_1–40_/Aβ_1–42_ ratio was not significantly associated with APOE4(+) status or disease severity at any of the eight time points (all *p* > 0.05).

We further compared the standard deviation of Aβ_1–42_ level between the APOE4(+) and APOE4(−) patients, and found that the APOE4(+) patients had smaller standard deviations at each time point (Table 3).

## Discussion

In this study, we investigated dynamic changes in plasma Aβ_1–40_ and Aβ_1–42_ concentrations from fresh blood samples at eight time points. The results showed that not every sample had a consistent increase in Aβ_1–40_ and Aβ_1–42_ level after 24 h (Aβ_1–40_: 62.5% and Aβ_1–42_: 87.5%). The effects of APOE4 genetic status on plasma Aβ_1–42_ level were observed only in sample measured within 30 min. Moreover, APOE4 genetic status did not affect plasma Aβ_1–40_ level at any time point. However, Aβ_1–40_ level was significantly higher in the patients with advanced stage dementia (CDR = 1 and CDR = 2) compared to those with mild stage dementia (CDR = 0.5).

### Effect of APOE4 Gene Status on Plasma Aβ_1–40_ and Aβ_1–42_ Levels

We used fresh plasma, and found significant differences in plasma Aβ_1–42_ at baseline (*p* = 0.031) and 0.5 h (*p* = 0.043) by APOE4 genetic status. However, no significant effects were noted at any other time points (Table 3), and no significant effects were observed in Aβ_1–40_ level at any time point. These findings are to some extent different to another study which examined plasma amyloid peptides in 19 non-demented participants immediately and after storage at room temperature for 24 and 48 h, respectively (Bibl et al., 2012). In their study, the authors reported that sample storage led to a significant loss of measurable amyloid peptide levels, and that this was most pronounced during the first 24 h of storage regardless of whether the distinct Aβ peptide species was Aβ_1–42_ or Aβ_1–40_ (Bibl et al., 2012). The differences between our study and Bibl’s study are that their samples came from non-demented participants, and they did not analyze changes in Aβ_1–42_ or Aβ_1–40_ between baseline and 24 h or control for APOE genetic status in their participants.

The effects of APOE4 genetic status on plasma Aβ_1–42_ level but not Aβ_1–40_ level in our samples indicates the possibility that there is an increase in the aggregation process of Aβ_1–42_ from the monomer to fibril form in APOE4 carriers. Similar findings have also been reported in a previous study, in which apo-lipoprotein E4 protein, mediated by the APOE4 gene, was shown to induce the aggregation of Aβ_1–42_ more specifically and rapidly than that of Aβ_1–40_ (Kang et al., 2015). The authors used a simple method to assess APOE4-mediated Aβ aggregation in physiological conditions using single gold nanoparticles based on localized surface plasmon resonance, which could be directly observed with a dark-field microscope or even by the naked eye, although some evidence of the biophysical properties of the interaction between apo-lipoprotein E4 protein and Aβ_1–42_ was unclear (Kang et al., 2015).

### Aβ_1–40_ and Aβ_1–42_ Levels in Relation to the Severity of AD

As mentioned, our results indicated that minor effects of APOE4 on plasma Aβ_1–42_ level and no significant effect on Aβ_1–40_ level. This is consistent with a previous longitudinal study which examined Aβ_1–40_ and Aβ_1–42_ levels in clinical trials (Donohue et al., 2015). In that study, there were no significant differences in Aβ_1–40_ and Aβ_1–42_ levels between AD patients who were and were not APOE4 carriers. However, there were significant differences in patients with mild cognitive impairment who had minor symptoms that were not sufficiently severe to be classified as AD (Donohue et al., 2015). The exact and detailed mechanisms by which APOE4 genetic status affects Aβ_1–40_ and Aβ_1–42_ levels should be clarified in other studies. However, the effects of APOE4 genetic status on our 0 and 0.5-h samples could more directly reflect the real situation of Aβ_1–42_ and Aβ_1–40_ in human blood because of the limited time from obtaining the venous samples.

In our samples, the patients with an advanced stage of dementia (CDR = 1 and CDR = 2) had increased Aβ_1–40_ levels compared to those with a very mild stage of dementia (CDR = 0.5). These findings could be, in part, attributed to the increased vascular contributions in advanced dementia (Yang et al., 2018), as the Aβ_1–40_ level has been reported to be higher in vascular amyloid deposition compared to Aβ_1–42_ (Iwatsubo et al., 1994; Gravina et al., 1995).

### Sample Storage

In this study, 87.5% of the AD patients had a higher mean Aβ_1–42_ level after 24 h (Figure 4), whereas only 62.5% of the AD patients had a higher mean Aβ_1–40_ level after 24 h (Figure 1). This finding is different to previous studies that reported a significant loss of measurable Aβ_1–42_ and Aβ_1–40_ peptide levels in their stored samples (Donohue et al., 2015; Kang et al., 2015). Donohue et al. (2015) examined the plasma concentrations of Aβ_1–42_ or Aβ_1–40_ after a longer storage time ranging from 0 to 1.8 years, and found that they declined significantly over time (−14.42 pg/ml Aβ_1–40_ per storage year, *p* < 0.001; −1.893 pg/ml Aβ_1–42_ per storage year, *p* = 0.003). In addition, Kang et al. (2015) reported that the loss of Aβ_1–40_ and Aβ_1–42_ was most pronounced during the first 24 h, in which the level of Aβ_1–40_ decreased from 267 ± 46 pg/ml at baseline to 190 ± 41 pg/ml at 24 h, and the level of Aβ_1–42_ decreased from 29 ± 4 pg/ml at baseline to 2 ± 4 pg/ml at 24 h. In order to avoid these declines or loss of Aβ_1–40_ and Aβ_1–42_, the authors recommended completing the measurements within 24 h after collecting the sample (Donohue et al., 2015). Compared to these studies, we provide more precise information with eight time points for both Aβ_1–42_ and Aβ_1–40_ levels within 24 h, and found that the changes in plasma Aβ_1–42_ and Aβ_1–40_ were dynamic and individualized.

### Measurement of Beta-Amyloid

The increase in mean plasma Aβ_1–42_ level at each time point in our AD patients may be due to several reasons. First, the Aβ_1–42_ aggregation process is continuous, from monomer, oligomer, protofibril, and eventually to fibril forms (Sinha and Lieberburg, 1999; Selkoe and Hardy, 2016). Compared to healthy subjects, the plasma of AD patients has been hypothesized to have a tendency to foster Aβ_1–42_ aggregation (Haass and Selkoe, 2007; Youn et al., 2019). Consistent with this hypothesis, the detected Aβ_1–42_ plasma levels increased from baseline to 24 h in this study. Second, the commercial kit that used to detect Aβ_1–42_ may not have only examined the monomer form of Aβ_1–42_, as the antibody in the kit may also have recognized the specific area of amyloid peptide, and amyloid peptide would continue the aggregation process to the formation of the fibril form. Third, Aβ_1–42_ may bind to carrier proteins such as apo-lipoprotein E and apo-lipoprotein J present in plasma (Matsubara et al., 1995; Zlokovic, 1996) that would possibly make the measurements difficult and result in a higher level. It is also possible that the commercial kit captured and detected oligomers in the sample. This would have resulted in an increase in the detected Aβ_1–42_ level at 24 h.

### Strengths and Limitations

This study has several strengths. First, we examined eight time points from 0 to 24 h and reported detailed changes in Aβ_1–42_ and Aβ_1–40_, and we also examined the effects of APOE4 genetic status and disease severity on plasma levels of Aβ_1–42_ and Aβ_1–40_. Second, we incubated all of the samples at 37°C before measurement to mimic the temperature of human blood in order to reduce possible confounding effects and approximate the actual level of Aβ_1–42_ and Aβ_1–40_. However, we do not know how beneficial this study design was, and the detailed effects and mechanisms of such design could be examined in another study. Third, we used the same protocol, commercial kits, and technician for all the examinations to avoid variabilities. There are also several limitations to this study. We reported changes in plasma Aβ_1–42_ and Aβ_1–40_ across all time points, however we did not examine the exact mechanisms, especially with regards to the possible effects of APOE genetic status, disease severity, or the possible bidirectional conversion between monomers and fibrils or oligomers. As there is currently no consensus on how best to evaluate dynamic changes in plasma Aβ_1–42_ and Aβ_1–40_, we chose the eight time points arbitrarily. These time points could be revised in future studies. In addition, our sample size was small, and further studies with a larger sample size comprehensively controlling for other confounding factors are warranted.

## Data Availability Statement

The raw data supporting the conclusions of this article will be made available by the authors, without undue reservation, to any qualified researcher.

## Ethics Statement

The studies involving human participants were reviewed and approved by the Kaohsiung Medical University Hospital IRB. The patients/participants provided their written informed consent to participate in this study.

## Author Contributions

Y-HY designed the study and wrote the manuscript. L-CH and S-WH recruited patients and collected samples. L-JH performed the ELISA analyses. All authors contributed to the article and approved the submitted version.

## Conflict of Interest

The authors declare that the research was conducted in the absence of any commercial or financial relationships that could be construed as a potential conflict of interest.

## Footnotes

1 http://www.pyrosequencing.com
